# Quantitative evaluation of hepatic steatosis using novel ultrasound technology normalized local variance (NLV) and its standard deviation with different ROIs in patients with metabolic-associated fatty liver disease: a pilot study

**DOI:** 10.1007/s00261-021-03394-0

**Published:** 2021-12-27

**Authors:** Yanan Zhao, Chao Zhang, Shaoyan Xu, Hui Zhang, Shumei Wei, Pengfei Huang, Lufei Zhang, Yik Ning Wong, Wen Xu, Pintong Huang

**Affiliations:** 1 Department of Ultrasound, Second Affiliated Hospital of Zhejiang University, School of Medicine, #88 Jiefang Road, Hangzhou, 310009 Zhejiang China; 2 Department of Hepatology, Second Affiliated Hospital of Zhejiang University, School of Medicine, Hangzhou, China; 3 Department of Pathology, Second Affiliated Hospital of Zhejiang University, School of Medicine, Hangzhou, China; 4 Canon Medical Systems China, Beijing, China

**Keywords:** Hepatic steatosis, Normalized local variance, Metabolic-associated fatty liver disease, Histopathology

## Abstract

**Purpose:**

The purpose of this study was to evaluate the diagnostic performance of novel ultrasound technology normalized local variance (NLV) and the standard deviation of NLV (NLV-SD) using different ROIs for hepatic steatosis in patients with metabolic-associated fatty liver disease (MAFLD) and to identify the factors that influence the NLV value and NLV-SD value, using pathology results as the gold standard.

**Methods:**

We prospectively enrolled 34 consecutive patients with suspected MAFLD who underwent percutaneous liver biopsy for evaluation of hepatic steatosis from June 2020 to December 2020. All patients underwent ultrasound and NLV examinations. NLV values and NLV-SD values were measured using different ROIs just before the liver biopsy procedure.

**Results:**

The distribution of hepatic steatosis grade on histopathology was 4/19/6/5 for none (< 5%)/ mild (5–33%)/ moderate (> 33–66%)/ and severe steatosis (> 66%), respectively. The NLV value with 50-mm-diameter ROI and NLV-SD value with 50-mm-diameter ROI showed a significant negative correlation with hepatic steatosis (spearman correlation coefficient: − 0.449, *p* = 0.008; − 0.471, *p* = 0.005). The AUROC of NLV (50 mm) for the detection of mild, moderate, and severe hepatic steatosis was 0.875, 0.735, and 0.583, respectively. The AUROC of NLV-SD (50 mm) for the detection of mild, moderate, and severe hepatic steatosis was 0.900, 0.745, and 0.603, respectively. NLV (50 mm) values and NLV-SD (50 mm) values between two readers showed excellent repeatability and the intraclass correlation coefficient (ICC) was 0.930 (*p* < 0.001) and 0.899 (*p* < 0.001). Hepatic steatosis was the only determinant factor for NLV value and NLV-SD value (*p* = 0.012, *p* = 0.038).

**Conclusion:**

The NLV (50 mm) and NLV-SD (50 mm) provided good diagnostic performance in detecting the varying degrees of hepatic steatosis with great reproducibility. This study showed that the degree of steatosis was the only significant factor affecting the NLV value and NLV-SD value.

## Introduction

Hepatic steatosis is characterized as abnormal accumulation of triglycerides (≥ 5%) in the liver [1]. Hepatic steatosis is a common histopathological feature of metabolic-associated fatty liver disease (MAFLD), alcoholic liver disease (ALD), chronic hepatitis B (CHB), and chronic hepatic C (CHC) infections [2]. Etiological factors which associated with fatty liver included diabetes, hepatitis, and drug toxicity [2]. Metabolic-associated fatty liver disease (MAFLD), formerly known as non-alcoholic fatty liver disease (NAFLD), is estimated to affect approximately 25% of the adult population worldwide, which endangered human health and imposed a huge economic burden on the society [3, 4]. MAFLD is closely associated with metabolic complications, such as obesity, diabetes, and dyslipidemia [5]. MAFLD pathologically encompasses the entire spectrum, ranging from isolated steatosis to severe hepatocellular injury with steatosis, and from inflammation and ballooning degeneration to advanced fibrosis [6]. The prognosis largely depends on the severity of histology. Although advanced fibrosis is still the strongest predictor of mortality in MAFLD patients [7, 8], the risk of disease progression and liver-related mortality in the early stages of the disease is also increasing [9]. It has been reported that significant steatosis can progress to non-alcoholic steatohepatitis and clinically significant fibrosis [9, 10]. Therefore, monitoring hepatic steatosis is of great significance in the early diagnosis, treatment, and follow-up of MAFLD patients.

Liver biopsy has traditionally been the gold standard for detecting and grading hepatic steatosis [5]. However, liver biopsy has several disadvantages such as it is invasive, it has high sampling error, and risk to surgery-related complications [5]. Therefore, a noninvasive method is desirable. Currently controlled attenuation parameter (CAP), derived from Transient Elastography (TE), attenuation imaging (ATI) using ultrasound (US), and proton density fat fraction (PDFF) measured by magnetic resonance imaging (MRI) have been developed as imaging tools for predicting hepatic steatosis and are with good diagnostic performance [11–14]. However, MRI-PDFF is expensive, time-consuming, and less available, which has no extensive clinical application. Meanwhile, CAP has poor diagnostic performance in detecting mild hepatic steatosis and the value is affected by etiology and metabolic factors [15]. Therefore, a noninvasive, cost-effective, and reliable imaging technique is needed to accurately assess hepatic steatosis.

**Fig. 1:**
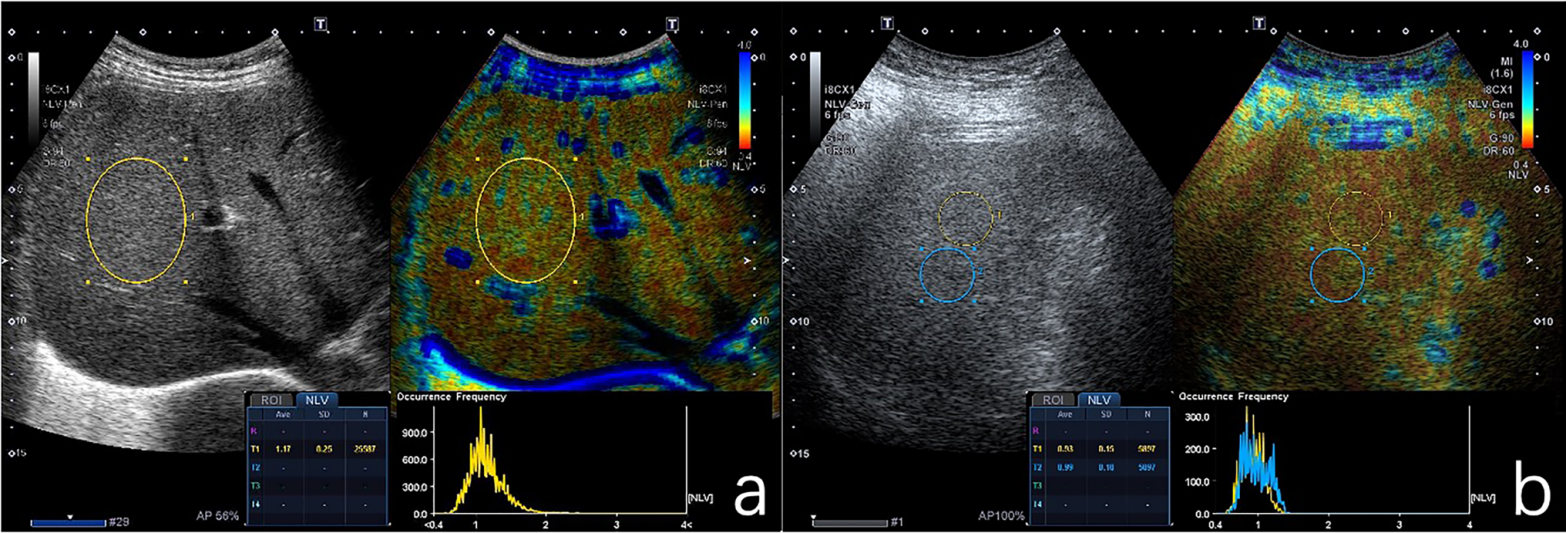
Representative images of NLV examination in a normal liver (**a**) and a fatty liver (**b**). One ROI (**a**) and two ROIs (**b**) were placed in the central portion of liver parenchyma, without artifacts or large hepatic vessels. Median NLV values obtained from the normal liver and fatty liver were 1.17 and 0.99, respectively

In recent years, a new technology normalized local variance (NLV), which is a quantitative tool to evaluate the intensity (brightness) and homogeneity (smoothness) of the target by performing regional analysis of the image, has been developed by Canon Medical Systems. Homogeneity of the liver is one of the most interesting parameters when evaluating diffuse liver disease [16]. This technique is based on the statistical analysis of differences between theoretical and actual echo amplitude from grayscale US images [17]. A tissue is often modeled as an aggregate of small sub-wavelength point scatters. Tiny objects smaller than the wavelength of the US beam cause scattering and interference of the beam, thereby creating a speckle pattern in the liver. Theoretically, the distribution of echo amplitude in the liver approaches the Rayleigh distribution. However, in a normal liver, the echo amplitude of the normal liver parenchyma deviates from the Rayleigh distribution due to the presence of blood vessels and bile duct walls, which are longer than the wavelength of the US beam and increase the heterogeneity of scattering, resulting in heterogeneous speckle patterns that deviate from the Rayleigh distribution. As hepatic steatosis progresses, small structures in the liver such as vessel walls would be masked by the increased echogenicity of the surrounding liver parenchyma. Therefore, the echo amplitude distribution of the fatty liver will be close to the theoretical Rayleigh distribution [17]. We considered that NLV technique could be used for the evaluation of hepatic steatosis through calculating the differences between theoretical and real echo amplitude distribution of the liver parenchyma (Fig. 1). This may help physicians to distinguish pathophysiologic properties without invasive methods such as liver biopsy.

The result of animal experiments [17] suggested that the NLV value had satisfactory diagnostic performance in the assessment of varying degrees of hepatic steatosis. The degree of hepatic steatosis was the only significant factor that affected the NLV value [17]. However, there is no further clinical research to explore the diagnostic performance of NLV in human hepatic steatosis. When using the normalized local variance (NLV) mode, the selection of region of interest (ROI) is not uniform [18, 19]. Some authors considered that the ROIs should be as large as possible and were placed on the liver parenchyma equivalent to other similar techniques; care was taken to avoid large hepatic vessels or artifacts [16, 18, 19]. Therefore, the purpose of this study was to evaluate the diagnostic performance of NLV for hepatic steatosis in MAFLD patients, with pathology as the gold standard. At the same time, seven sizes of circular ROI were selected for measurement to explore whether the selection of ROI size is an influencing factor for NLV assessment of hepatic steatosis.

## Materials and methods

### Patients

This prospective, single-center study was approved by the Institutional Review Board of the Second Affiliated Hospital of Zhejiang University School of Medicine. Written informed consent was obtained from all patients. Patients who were suspected to have MAFLD and who were referred for liver biopsy to evaluated the etiology and disease activity were consecutively enrolled from the department of gastroenterology and hepatology between July 2020 and December 2020. The inclusion criteria were as follows: male or female aged 20 or older; hepatic steatosis detected by B-mode US and met MAFLD diagnostic criteria [6]. The exclusion criteria were the followings: patients with significant bleeding risk (platelet prothrombin < 80,000/μL and prothrombin time > 20 s); patients with a history of alcohol use (pure alcohol above 30 g/day for male, 20 g/day for female); patients with viral hepatitis, malignant liver tumor, common bile duct stone, and jaundice; patients with primary biliary cholangitis, primary sclerosing cholangitis, and autoimmune hepatitis.

### Normalized local variance (NLV) examination

**Fig. 2:**
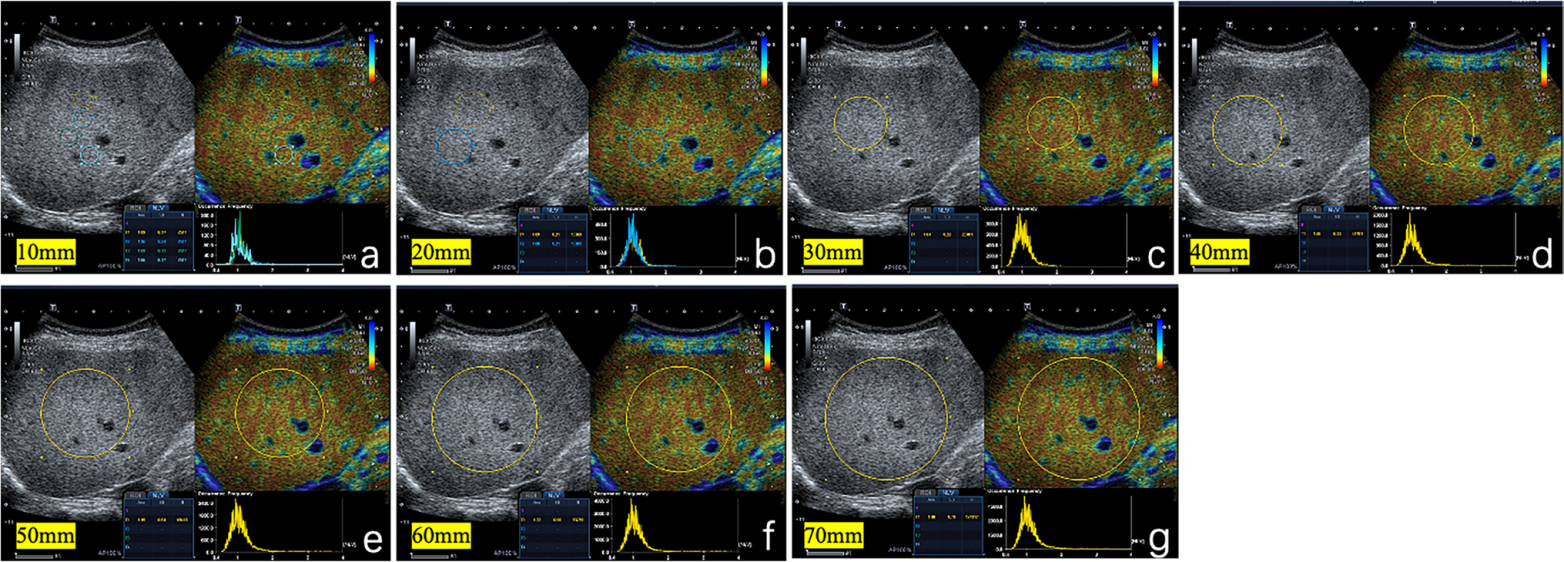
Evaluation of liver parenchyma using NLV with different sizes of ROIs in a 45-year-old female patient. Seven different sizes of ROIs were placed in the liver parenchyma, not including artifacts. 10 mm ROIs to 40 mm ROIs were placed on the liver parenchyma. Blood vessels were avoided as much as possible (**a-d**); 50 mm ROI was placed in the middle liver parenchyma and 10 mm under the liver capsule (**e**); 60 mm circular ROI, 70 mm circular ROI were placed to included maximal liver parenchyma(**f–g**). NLV, normalized local variance, *NLV-SD* standard deviation of normalized local variance; *ROI* region of interest

All Normalized Local Variance (NLV) examinations were performed by one radiologist (with six years of experience in abdominal US imaging) prior to liver biopsy using an US scanner with a convex transducer (PVI-475BX, 4 MHz; Aplio i900; Canon Medical Systems, Tochigi, Japan). All patients fasted for at least four hours before the examination. The patient was in the supine position and the right arm was extended above the head to stretch the intercostal muscles and obtain the proper scanning window during the examination. First, liver parenchyma was evaluated on B-mode images to detect any focal liver lesion. After that, NLV mode was activated, and examinations were performed in the right lobe of the liver through an intercostal window with the transducer perpendicular to the skin surface while the patient held his or her breath. In NLV mode, four 10 mm circular ROIs were placed in the liver parenchyma at different depth from 10 mm under the liver capsule that avoided the subcapsular area, hepatic vessels, and artifacts. On the same US image, replacing the 10 mm ROI, two 20 mm circular ROIs, one 30 mm circular ROI, one 40 mm circular ROI, one 50 mm circular ROI, one 60 mm circular ROI, and one 70 mm circular ROI were placed, respectively, on the liver parenchyma where the location is free of artifacts (Fig. 2). Notice that 10 mm circular ROI, 20 mm circular ROI, 30 mm circular ROI and 40 mm circular ROI were placed on the liver parenchyma that avoided blood vessels as much as possible; 50 mm circular ROI was placed in the middle of the US image and 10 mm under the liver capsule no matter whether certain blood vessels were included; 60 mm circular ROI, 70 mm circular ROI included certain blood vessels with maximal liver parenchyma. The NLV value was automatically calculated and displayed on the lower left position of the screen. In the result area, average value and standard deviation of NLV, and sampling number within each ROI for calculating NLV were displayed. In the graph area next to the results, a histogram was displayed, which contained the NLV value on the x-axis and occurrence frequency on the y-axis. The NLV examination was performed from four different US images and the median of different ROIs from all four US images were used for the analysis. Additional analysis was performed according to primary results, NLV (50 mm) value and NLV-SD (50 mm) value were measured by two radiologists (with six and three years of experience in abdominal US imaging) who were blinded with each other and pathological results.

### Biochemical and histopathologic examination

We documented patients’ background data and blood test data before liver biopsy. Background of patient included age, gender, height (cm), weight (kg), waist circumference (cm), BMI (kg/m^2^), presence of diabetes mellitus or hypertension. Fasting blood test items included platelet counts (PLT) (109/L), aspartate aminotransferase (AST) (IU/L), alanine aminotransferase (ALT) (IU/L), γ-glutamyl transpeptidase (GGT) (IU/L), fasting blood glucose (mmol/L), triglycerides (TG) (mmol/L), total cholesterol (TC) (mmol/L), total bilirubin (TBIL) (μmol/L), alkaline phosphatase (ALP) (U/L), albumin (g/L), low density lipoprotein cholesterol (LDL-C) (mmol/L), high density lipoprotein cholesterol (HDL-C) (mmol/L), plasma urea nitrogen (BUN) (mmol/L), uric acid (UA) (μmol/L), C-reactive protein (CRP) (mg/L).

Percutaneous liver biopsy using a semi-automatized needle (NS18/16, NS16/16, GALLINI S R L.) was performed after Normalized Local Variance (NLV) examination. Biopsy area was performed in liver segment V or VIII. A liver specimen of more than 1.5 mm with at least nine portal tracts was considered adequate for evaluation. Liver biopsy specimens were fixed in formalin and embedded in paraffin. Subsequently, 5 μm-thick tissue slices were cut and stained with hematoxylin–eosin and Masson trichrome. All histopathologic examinations were analyzed by two expert pathologists (with 15 years and 5 years of experiences in liver pathology) who were blinded to the US examination results. The NAFLD Activity Score (NAS) was used to evaluate the pathological parameters of MAFLD (steatosis, intralobular inflammation, ballooning) [1]. The degree of steatosis (S) was graded on a four-point scale as follows: S0 (< 5%, none), S1 (5–33%, mild), S2 (> 33–66%, moderate), and S3 (> 66%, severe). Lobular inflammation (I) was graded from score 0 to 3 as follows: I0 (no foci), I1 (< 2 foci per 200 × filed), I2 (2–4 foci per 200 × filed), I3 (> 4 foci per 200 × filed) (Table 1). Hepatocyte ballooning degeneration (B) was graded from score 0 to 2 as follows: B0 (none), B1 (few balloon cells), B2 (many cells or prominent ballooning) (Table 1). Then the NAFLD activity score (NAS) was calculated as the sum of the scores of steatosis (S, 0–3), lobular inflammation (I, 0–3), and hepatocyte ballooning (B, 0–2), which ranged from 0 to 8. The fibrosis stage (F) was evaluated on a five-point scale from F0 to F4 according to Brunt Classification [20], as follows: F0 (no fibrosis), F1 (fibrosis near lobule center), F2 (fibrosis near lobule center and periportal fibrosis), F3 (bridging fibrosis),F4 (cirrhosis) (Table 1).

**Table 1:** Baseline characteristics of the 34 patients with MAFLD

Parameters	Patients (*n* = 34)
Age (years, mean ± SD) [range]	55.2 ± 10.4 [27–72]
Sex (*n*, male:female)	12:22
Diabetes (*n*, yes:no)	7:27
Hypertension (*n*, yes:no)	19:15
BMI (kg/m^2^, mean ± SD) [range]	27.3 ± 4.1 [20.3–36.3]
Waist circumference (cm,mean ± SD) [range]	97.1 ± 9.9 [78–121]
Liver biochemistry	
AST (IU/L, mean ± SD) [range]	31.7 ± 22.2 [14–139]
ALT (IU/L, mean ± SD) [range]	40.2 ± 37.5 [13–219]
γ-GT (IU/L, mean ± SD) [range]	48.5 ± 47.9 [9–221]
ALP (IU/L, mean ± SD) [range]	90.0 ± 25.2 [33–153]
TBIL (μmol/L, mean ± SD) [range]	17.5 ± 23.8 [5.3–148]
Lipid profile	
Cholesterol (mg/dL, mean ± SD) [range]	5.6 ± 0.9 [3.7–7.6]
HDL-C (mg/dL, mean ± SD) [range]	1.2 ± 0.3 [0.8–2.1]
LDL-C (mg/dL, mean ± SD) [range]	3.1 ± 0.7 [1.8–4.4]
Triglycerides (mg/dL, mean ± SD) [range]	1.9 ± 0.7 [0.7–4.2]
Albumin (g/dL, mean ± SD) [range]	43.6 ± 2.8 [37.2–50.4]
Platelets (× 109/L, mean ± SD) [range]	224 ± 69 [74–405]
Blood glucose (mg/dL, mean ± SD) [range]	5.9 ± 1.3 [4.0–10.0]
BUN (mmol/L, mean ± SD) [range]	4.5 ± 1.2 [2.4–7.5]
UA (μmol/L, mean ± SD) [range]	378.0 ± 98.6 [220–607]
Depth (cm)	2.0 ± 0.4 [1.2–3.2]
Degree of steatosis (%)	
S0 (none, < 5%)	4 (11.8)
S1 (mild, 5–33%)	19 (55.9)
S2 (moderate, 33–66%)	6 (17.6)
S3 (severe, > 66%)	5 (14.7)
Intralobular Inflammation	
I0 (None)	6 (17.6)
I1 (Mild)	20 (58.8)
I2 (Moderate)	6 (17.6)
I3 (Severe)	2 (5.9)
Ballooning degeneration	
B0 (None)	23 (67.6)
B1 (Few balloon cells)	8 (23.5)
B2 (Many cells/prominent ballooning)	3 (8.8)
Grade of fibrosis (%)	
F0	18 (52.9)
F1	10 (29.4)
F2	5 (14.7)
F3	1 (2.9)
F4	0 (0)

*BMI* body mass index, *AST* aspartate aminotransferase, *ALT* alanine aminotransferase, *γ-GT* γ-glutamyl transpeptidase, *ALP* alkaline phosphatase, TBIL; *HDL-C* high density lipoprotein cholesterol, *LDL-C* low density lipoprotein cholesterol, *BUN* plasma urea nitrogen, *UA* uric acid

### Statistical analysis

Statistical analysis was performed using SPSS software version 17 (IBM Corp., Armonk, NY, USA), and Medcalc software version 12.1.00 (MedCalc Software, Mariakerke, Belgium). Continuous data were expressed as mean ± standard deviation or median ± interquartile range, and count data were presented as absolute number or percentages. Spearman rank correlation coefficient was used to evaluate the correlation between NLV, NLV-SD and histological grade of steatosis. Continuous variables were compared using the Kruskal–Wallis test, and categorical variables were evaluated using the Chi-square test or the Fisher’s exact test. Intra-observer reproducibility of NLV values and NLV-SD values were assessed using intraclass correlation coefficients (ICCs) and Bland Altman analysis. The diagnostic performance of the NLV value and NLV-SD value in the detection of hepatic steatosis was calculated by the receiver operating characteristic (ROC) curve analysis. Univariate and multivariate linear analysis were conducted to determine the factors that affected the NLV value. All significance tests were two-sided, descriptive levels (*p* values) lower than 0.05 were considered statistically significant.

## Results

### Baseline characteristics

During the study period, a total of 46 consecutive patients with fatty liver revealed by conventional US or abdominal CT were referred for liver biopsy at our institution. NLV examination prior to liver biopsy was performed. 12 patients with chronic hepatitis B were excluded from the study and our final study population comprised a total of 34 patients. The mean (± standard deviation) values for age and body mass index (BMI) were 55.2 years ± 10.4 and 27.3 kg/m^2^ ± 4.1, respectively. The participants’ baseline demographic, biochemical, and histologic data are summarized in Table 1.

### Hepatic steatosis according to the steatosis grade

**Fig. 3:**
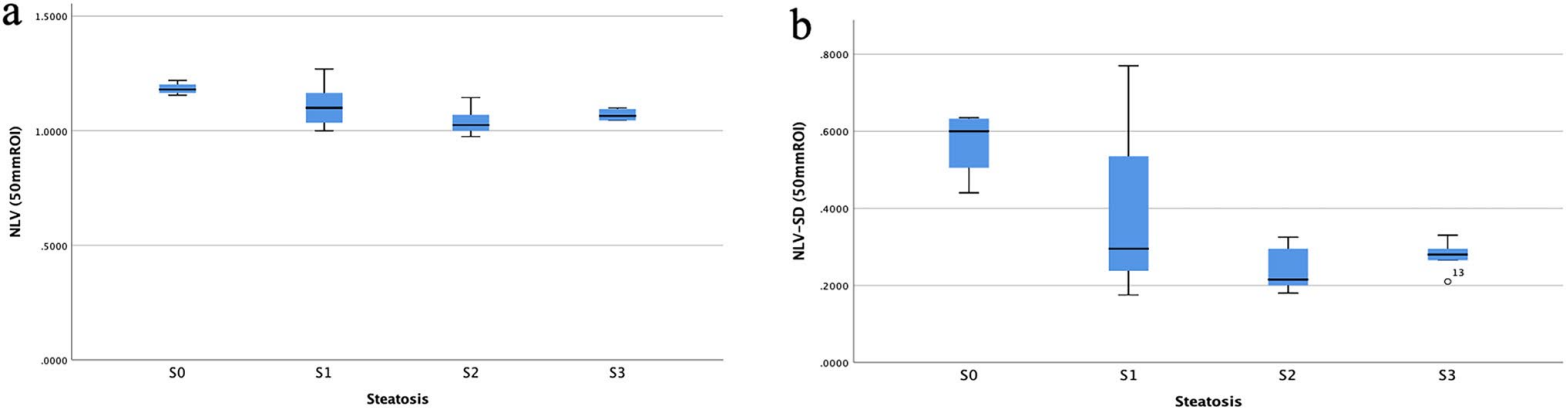
The distribution of NLV (50 mm) value and NLV-SD (50 mm) value according to the hepatic steatosis grade on histopathology. The hepatic steatosis grade: S0 (*n* = 4), S1 (*n* = 19), S2 (*n* = 6), and S3 (*n* = 5). Median values of NLV (50 mm) value and NLV-SD (50 mm) value for each steatosis grade are given (**a**, **b**). The central box represents values for the lower to upper quartile (25–75 percentile). The middle line represents the median. A line extends from the minimum to the maximum value (range). Excluding outlying values, which are displayed as separate points. *NLV* normalized local variance, *NLV-SD* standard deviation of normalized local variance, *ROI* region of interest

The median values of different circular ROIs from 10 to 70 mm obtained from NLV are summarized in Table 2. The results showed that the NLV (50 mm) values and NLV-SD (50 mm) values were significantly different among the patients with different grades of hepatic steatosis. Boxplots of NLV (50 mm) values and NLV-SD (50 mm) values versus hepatic steatosis are shown in Fig. 3. The spearman correlation showed that NLV (50 mm) values and NLV-SD (50 mm) values had significant negative correlation with hepatic steatosis (the correlation coefficient is − 0.449, *p* = 0.008; − 0.471, *p* = 0.005) (Table 3).

**Table 2:** NLV value and NLV-SD value of different ROIs in the diagnosis of hepatic steatosis

Size (mm)	S0 (*n* = 4)	S1 (*n* = 19)	S2 (*n* = 6)	S3 (*n* = 5)	*Z* value	*p* value
NLV (10)	1.00 (0.96–1.05)	1.00 (0.94–1.15)	1.00 (0.94–1.06)	1.03 (1.00–1.06)	3.30	0.348
NLV-SD (10)	0.17 (0.15–0.20)	0.17 (0.14–0.23)	0.16 (0.14–0.20)	0.17 (0.15–0.18)	1.94	0.585
NLV (20)	1.05 (1.01–1.09)	1.04 (0.98–1.17)	1.01 (0.98–1.10)	1.06 (1.03–1.13)	5.14	0.162
NLV-SD (20)	0.22 (0.20–0.27)	0.20 (0.16–0.25)	0.18 (0.16–0.20)	0.20 (0.18–0.22)	8.48	0.037*
NLV (30)	1.04 (1.00–1.07)	1.04 (0.98–1.18)	1.03 (0.98–1.14)	1.04 (1.02–1.05)	2.17	0.539
NLV-SD (30)	0.23 (0.18–0.28)	0.21 (0.17–0.28)	0.20 (0.17–0.28)	0.20 (0.19–0.22)	1.81	0.612
NLV (40)	1.08 (1.03–1.15)	1.05 (0.99–1.20)	1.02 (0.97–1.13)	1.07 (1.01–1.13)	5.00	0.172
NLV-SD (40)	0.31 (0.20–0.41)	0.24 (0.18–0.43)	0.20 (0.17–0.27)	0.24 (0.19–0.30)	7.15	0.067
NLV (50)	1.18 (1.16–1.22)	1.10 (1.00–1.27)	1.04 (0.98–1.15)	1.07 (1.05–1.10)	9.31	0.025*
NLV-SD (50)	0.57 (0.44–0.64)	0.38 (0.18–0.77)	0.24 (0.18–0.33)	0.28 (0.21–0.33)	10.10	0.018*
NLV (60)	1.19 (1.14–1.25)	1.13 (1.00–1.35)	1.05 (0.98–1.20)	1.11 (1.03–1.21)	6.10	0.107
NLV-SD (60)	0.60 (0.51–0.71)	0.49 (0.18–1.31)	0.26 (0.18–0.42)	0.45 (0.20–0.89)	6.25	0.100
NLV (70)	1.18 (1.13–1.21)	1.13 (1.00–1.40)	1.06 (0.97–1.19)	1.12 (1.02–1.22)	5.03	0.170
NLV-SD (70)	0.58 (0.51–0.64)	0.54 (0.19–1.24)	0.29 (0.18–0.44)	0.47 (0.19–0.80)	5.31	0.150

*p < 0.05

**Table 3:** Correlation between NLV (50 mm) value, NLV-SD (50 mm) value, and pathological parameters

	Steatosis	Inflammation	Ballooning	Fibrosis
NLV (50 mm)	− 0.449*	− 0.285	− 0.163	− 0.226
*p* value	0.008	0.102	0.358	0.198
NLV-SD (50 mm)	− 0.471*	− 0.239	− 0.197	− 0.272
*p* value	0.005	0.173	0.263	0.120

*p < 0.05

### Factors affecting the NLV (50 mm) value and NLV-SD (50 mm) value

The factors affecting the NLV (50 mm) value and NLV-SD (50 mm) value are summarized in Tables 4 and 5. We included BMI and factors that were significantly less than 0.1 in the univariate regression analysis for multivariate linear regression analysis. According to the univariate analysis, the degree of steatosis and AST were associated with NLV (50 mm) value. However, the degree of steatosis was the only significant factor determining the NLV (50 mm) value (*p* = 0.012) according to the multivariate linear regression analysis (Table 4). According to the univariate analysis, the degree of steatosis, the degree of ballooning degeneration and the grade of fibrosis were associated with NLV-SD (50 mm) value. However, the degree of steatosis was the only significant factor determining the NLV-SD (50 mm) value according to the multivariate linear regression analysis (*p* = 0.003) (Table 5).

**Table 4:** Factors associated with NLV (50 mm) value

Characteristic	Univariate analysis			Multivariate analysis		
	Coefficient	95% CI	*p* value	Coefficient	95% CI	*p* value
BMI	− 0.005	− 0.011 to 0.002	0.178	–	–	–
Diabetes mellitus	− 0.038	− 0.105 to 0.030	0.265			
Hypertension	− 0.039	− 0.093 to 0.015	0.154			
AST	5.776E-6	− 0.001 to 0.001	0.993	–	–	–
ALT	0.000	− 0.001 to 0.001	0.596			
Degree of steatosis	− 0.038	− 0.066 to − 0.009	0.012^*^	− 0.038	− 0.066 to 0.009	0.012*
Intralobular Inflammation	− 0.027	− 0.062 to 0.008	0.130			
Ballooning Degeneration	− 0.024	− 0.066 to 0.018	0.260			
Grade of fibrosis	− 0.017	− 0.053 to 0.018	0.316			
VAS score	− 0.018	− 0.051 to 0.015	0.279			

*p < 0.05

**Table 5:** Factors associated with NLV-SD (50 mm) value

Characteristics	Univariate analysis			Multivariate analysis		
	Coefficient	95% CI	*p* value	Coefficient	95% CI	*p* value
BMI	− 0.008	− 0.022 to 0.007	0.284	–	–	–
Diabetes mellitus	− 0.080	− 0.224 to 0.064	0.266			
Hypertension	− 0.091	− 0.206 to 0.024	0.116			
AST	0.000	− 0.003 to 0.002	0.765			
ALT	− 0.001	− 0.002 to 0.001	0.516			
Degree of steatosis	− 0.095	− 0.154 to 0.036	0.003*	− 0.095	− 0.154 to 0.036	0.003**
Intralobular Inflammation	− 0.068	− 0.142 to 0.007	0.072	–	–	–
Ballooning Degeneration	− 0.081	− 0.168 to 0.007	0.069	–	–	–
Grade of fibrosis	− 0.067	− 0.135 to 0.000	0.050	–	–	–

*p < 0.05; **p < 0.01

### Intra-observer variability of NLV measurements

**Fig. 4:**
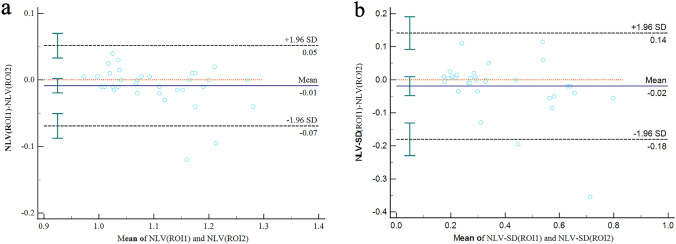
Bland–Altman Plot of Reader 1 vs Reader 2 for NLV (50 mm) value and NLV-SD (50 mm) value (**a**, **b**), showing line of mean bias (− 0.01, − 0.02) and the 95% tolerance limits about zero-bias line. NLV, normalized local variance; *NLV-SD* standard deviation of normalized local variance

The mean of the median NLV (50 mm) values by the two readers were 1.10 ± 0.08 and 1.10 ± 0.09, respectively, while with mean values of 0.36 ± 0.17 and 0.38 ± 0.20 for NLV-SD (50 mm) (Fig. 4). The intraclass correlation coefficient (ICC) were 0.930 (*p* < 0.001) and 0.899 (*p* < 0.001) for NLV (50 mm) and NLV-SD (50 mm) between the two readers, which showed excellent repeatability of NLV values and NLV-SD values. The Bland Altman plot (Fig. 4) showed a large number of values near the zero-bias line, and a very slight positive bias of − 0.01 and − 0.02 in NLV (50 mm) value and NLV-SD (50 mm) value.

### Diagnostic performance of NLV (50 mm) and NLV-SD (50 mm) in the grading of hepatic steatosis

**Fig. 5:**
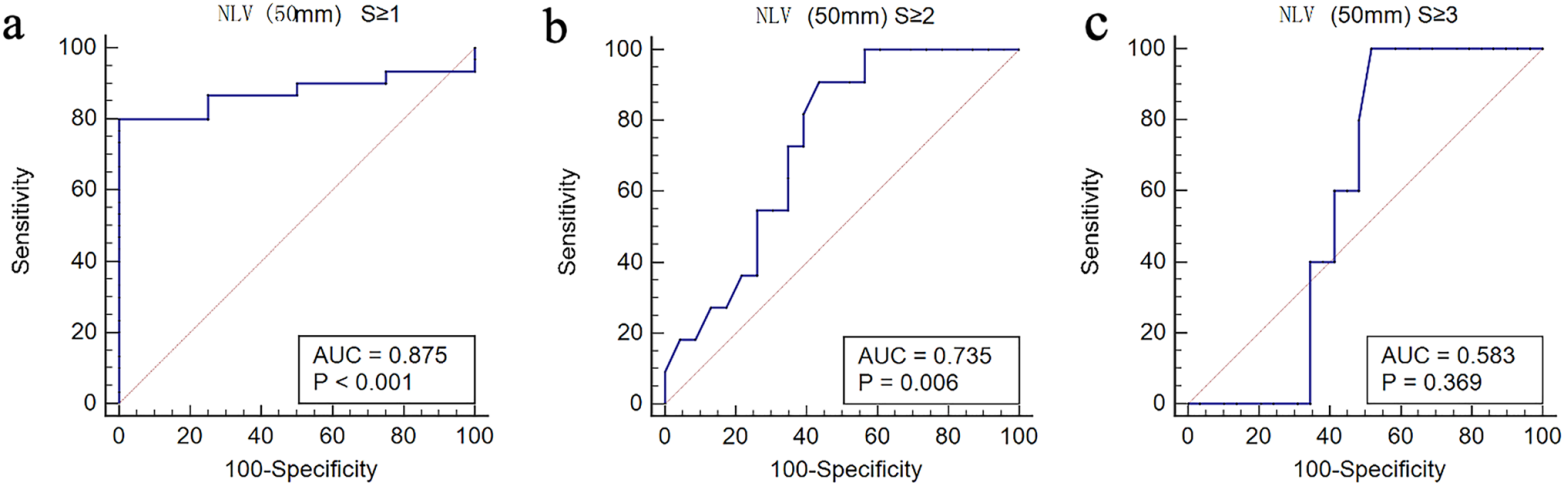
Receiver operating curve for the diagnostic performance of the NLV (50 mm) value in the diagnosis of the presence of hepatic steatosis (S1 to S3) (**a**), the presence of moderate-to-severe steatosis (S2 to S3) (**b**), and the presence of severe steatosis (S3) (**c**). The areas under the ROC curve were 0.875 (95% CI 0.716–0.963, *p* < 0.001), 0.735 (95% CI 0.556–0.871, *p* = 0.006), and 0.583 (95% CI 0.402–0.749, *p* = 0.369) for the diagnosis of steatosis (S1–S3), moderate-to-severe steatosis (S2–S3), and severe steatosis (S3), respectively. *NLV* normalized local variance, *ROI* region of interest

**Fig. 6:**
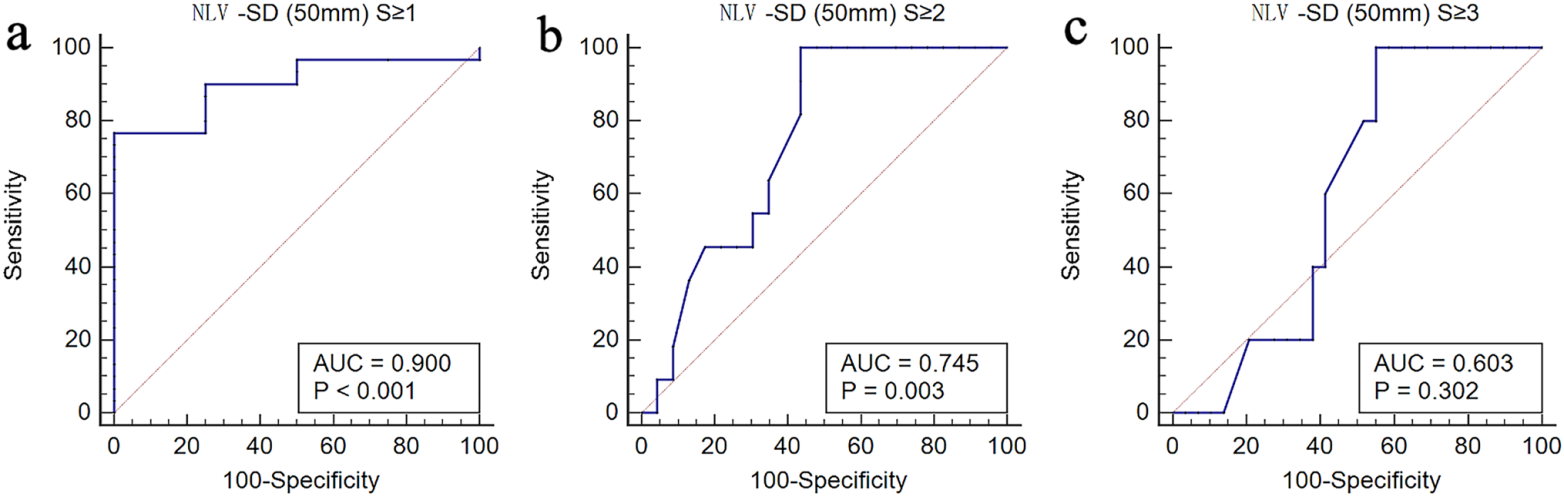
Receiver operating curve for the diagnostic performance of the NLV-SD (50 mm) value in the diagnosis of the presence of hepatic steatosis (S1 to S3) (**a**), the presence of moderate-to-severe steatosis (S2 to S3) (**b**), and the presence of severe steatosis (S3) (**c**). The areas under the ROC curve were 0.900 (95% CI 0.748, 0.976, *p* < 0.001), 0.745 (95% CI 0.567–0.878, *p* = 0.003), and 0.603 (95% CI 0.422–0.766, *p* = 0.302), respectively. *NLV-SD* standard deviation of normalized local variance, *ROI* region of interest

The area under curve (AUC) and optimal cut-off values with the corresponding sensitivities and specificities of the NLV (50 mm) value and NLV-SD (50 mm) value for detecting each grade of hepatic steatosis are summarized in Tables 6 and 7. The AUC of NLV value (50 mm) was 0.875, 0.735 and 0.583 for detecting S ≥ 1, S ≥ 2 and S ≥ 3, respectively, with the sensitivity and specificity of 80.0% and 100.0%, 90.9% and 56.5%, 100.0% and 48.3% for detecting S ≥ 1, S ≥ 2 , and S ≥ 3, respectively. The optimal cut-off for NLV values (50 mm) were 1.145, 1.1 and 1.1 for S ≥ 1, S ≥ 2 , and S ≥ 3, respectively (Fig. 5). The AUC of NLV-SD value (50 mm) was 0.900, 0.745 and 0.603 for detecting S ≥ 1, S ≥ 2 , and S ≥ 3, respectively, with the sensitivity and specificity of 76.7% and 100.0%, 100.0% and 56.5%, and 100.0% and 44.8% for detecting S ≥ 1, S ≥ 2 , and S ≥ 3, respectively. The optimal cut-off for NLV-SD (50 mm) values were 0.365, 0.33 and 0.33 for S ≥ 1, S ≥ 2 , and S ≥ 3, respectively (Fig. 6).

**Table 6:** Diagnostic performance of NLV (50 mm) value in detecting grade of steatosis

Grade of hepatic steatosis	Cutoff value	AUC (95% CI)	Sensitivity (%) (95% CI)	Specificity (%) (95% CI)	Positive predictive value (%)	Negative predictive value (%)
S0 vs S1, S2, S3	1.145	0.875 (0.716, 0.963)	80.0 (61.4,92.3)	100 (39.8,100.0)	100	92.1
S0, S1 vs S2, S3	1.1	0.735 (0.556,0.871)	90.9 (58.7,99.8)	56.5 (34.5,76.8)	47.3	93.6
S0, S1, S2 vs S3	1.1	0.583 (0.402,0.749)	100.0 (47.8,100.0)	48.3 (29.4,67.5)	45.3	100.0

**Table 7:** Diagnostic performance of NLV-SD (50 mm) value in the detection of hepatic steatosis

Grade of hepatic steatosis	Cutoff value	AUC (95% CI)	Sensitivity (%) (95% CI)	Specificity (%) (95% CI)	Positive predictive value (%)	Negative predictive value (%)
S0 vs S1, S2, S3	0.365	0.900 (0.748, 0.976)	76.7 (57.7,90.1)	100.0 (39.8,100.0)	100	90.9
S0, S1 vs S2, S3	0.33	0.745 (0.567,0.878)	100.0 (71.5,100.0)	56.5 (34.5,76.8)	49.6	100.0
S0, S1, S2 vs S3	0.33	0.603 (0.422,0.766)	100.0 (47.8,100.0)	44.8 (26.4,64.3)	43.7	100.0

## Discussion

To our knowledge, there was no previous study to investigate the diagnostic performance of NLV technique and its evaluation of hepatic steatosis in MAFLD patients. With histopathologic examination as a reference standard in our study, NLV and NLV-SD provided good diagnostic performance in evaluating the degree of hepatic steatosis. Our study also provided NLV (50 mm) value and NLV-SD (50 mm) value as the best advice for measurement selection when using the NLV technique. This study showed NLV value and NLV-SD value were significantly negative correlated with the degree of hepatic steatosis and had excellent intra-observer repeatability. The AUCs of the NLV values (50 mm) for the detection of hepatic steatosis grade ≥ S1, ≥ S2, ≥ S3 in MAFLD patients were 0.875, 0.735 ,and 0.583, respectively. The AUCs of the NLV-SD (50 mm) values for the detection of hepatic steatosis grade ≥ S1, ≥ S2, ≥ S3 in MAFLD patients were 0.900, 0.745, and 0.603, respectively. The degree of steatosis was the only significant factor determining the NLV value and NLV-SD value according to the univariate and multivariate analysis. This result was in accordance with the results of previous animal studies reporting that the degree of hepatic steatosis was the only significant factor that affected the NLV value [17].

An early and accurate detection of hepatic steatosis is of great importance because MAFLD is associate with several metabolic comorbidities, and may progress into more advanced stages during the disease course [21]. NLV based on US can be performed repeatedly during the disease course with its advantage of low cost, convenience and no risk to the patient. Clinically, moderate steatosis (triglyceride content > 33%), which is defined as significant steatosis, is associated with fibrosis progression in patients with NAFLD [9]. The results of this study showed that NLV is a valuable biomarker, showing the AUC of 0.9 for detection of ≥ mild steatosis, 0.745 for detection of ≥ moderate steatosis. Even through the diagnostic performance of NLV and NLV-SD in detecting of severe steatosis was reduced, the result may be related to the pathological similarity between moderate and severe steatosis. Available evidence showed increased risk of poor graft outcome in moderate-severe fatty liver [22]. Liver grafts with < 30% steatosis can be safely used for liver transplantation [23]. Our results showed good diagnostic performance for detection of more than moderate steatosis (≥ 33%), which can be used in selecting available donor liver grafts in the future.

NLV technique is a novel US imaging technique to analyze the tissue homogeneity in the liver, which is similar with another technique called acoustic structure quantification (ASQ) [18]. There has been no consensus on the size and placement of ROI when using these techniques, which leads to inaccuracies in data collection and different results [16, 18, 19]. In our study, NLV images were obtained in the right lobe of the liver through an intercostal window with the transducer perpendicular to the skin, the image depth is subjected to include the whole right liver parenchyma and avoid large blood vessels as much as possible. We chose different sizes of ROI and placed them in different positions of the NLV images. 10 mm circular ROI to 40 mm circular ROI were placed in the liver parenchyma, while avoiding large blood vessels and lateral shadows as much as possible. 50 mm circular ROI to 70 mm circular ROI were placed at least 10 mm under the liver capsule in the center of US image to avoid lateral shadows as much as possible, regardless of whether it avoids large blood vessels. Our results demonstrated that only NLV value and NLV-SD value of 50 mm circular ROI were significantly negative correlated with the degree of hepatic steatosis. However, if ROIs were too small or placed in a position that does not contain obvious blood vessels at all, the NLV value could not show the difference of echo amplitude distribution. This finding is not completely consistent with previous studies that ROIs should avoid large blood vessels [16, 18, 19]. We found 50 mm ROI was suitable when it is neither too small to avoid certain blood vessels, nor too large to include 10 mm parenchyma under the liver capsule, lateral shadows, and posterior large blood vessels. Our research results can provide evidence for more accurate use of this technology in clinical or future researches.

Our study showed that hepatic steatosis is the only factor that significantly causing changes in NLV value and NLV-SD value. The increase in the number and size of fat droplets may result in homogenization of US images, which is easy to understand [24]. Fibrosis is another pathological progression stage of diffuse liver disease in MAFLD [6]. The changes in liver texture may also affect the heterogeneity of US images. Several studies have demonstrated that in patients with CHB or CHC, steatosis is an independent risk factor associated with severe fibrosis [25–27]. Previous studies have shown steatosis and fibrosis were significant factors that affected some imaging techniques such as ASQ and attenuation imaging (ATI) [18, 25]. Another study found fibrosis stage had no statistically significant correlation of measurement parameters with steatosis grade [14]. In this group, we found that there was no difference in the NLV values and NLV-SD values among the stage of hepatic fibrosis. The distribution of the stage of hepatic fibrosis was uneven in this group of cases, with one case of advanced fibrosis (F3), others of no (F0) or early fibrosis (F1 and F2). Therefore, the sample size was too small to compare with the NLV values and NLV-SD values according to different stages of hepatic fibrosis. Meanwhile, it also demonstrated that patients with lower fibrosis stages (< F2) should not have affected the diagnostic accuracy of NLV and NLV-SD in the diagnosis of hepatic steatosis in MAFLD patients. Further studies should include more cases with even distribution of stages of fibrosis with the same level of steatosis to discuss the effect of hepatic fibrosis on NLV technique. The relatively narrow range of the NLV (50 mm) value (mostly between 0.98 and 1.27) in different degree of hepatic steatosis may also indicate even mild steatosis can make homogeneous liver parenchyma and decrease the NLV values. This coincided with the results of previous animal experiments [17].

There are some limitations in this study. First, it is a single-center study, multi-center studies will be needed to confirm the significance of NLV in the degree of hepatic steatosis. Secondly, the sample size of this study was relatively small and the distribution of hepatic steatosis was deviated: the number of cases with no (*n* = 4), moderate (*n* = 6), or severe (*n* = 5) steatosis was relatively small compared to the number of cases with mild steatosis (*n* = 19). More samples with uniform distribution of different degree of steatosis will be needed in the future study. Thirdly, there were few cases of severe fibrosis or cirrhosis in this study, the exact effect of advanced fibrosis on the NLV value and NLV-SD value could not be investigated. Fourthly, all NLV examinations were performed by the same radiologist and the intra-observer reproducibility of NLV was evaluated in one image between two radiologists, which would cause sample errors. It was difficult to perform multiple measurements of NLV with two operators in MAFLD patients due to patient examination time limit. Therefore, to evaluate the reproducibility of the measurement, further studies with two or more operators are required. Fifth, the selected cut-off values of NLV and NLV-SD for steatosis grades greater than S2 and S3 were the same, which would limit real-life operation in clinical settings. However, it is sufficient to identify more than moderate degree of steatosis in the evaluation of donor liver grafts for liver transplantation.

## Conclusion

In conclusion, the NLV value and NLV-SD value demonstrated a satisfactory diagnostic performance in the degree of hepatic steatosis in patients with MAFLD. NLV (50 mm) value and NLV-SD (50 mm) value were the best choices for measurement selection. The NLV (50 mm) value and NLV-SD (50 mm) value showed a significantly negative correlation with the degree of hepatic steatosis with excellent intra-observer repeatability. In this study, the degree of steatosis was the only factor that significantly affected the NLV value and NLV-SD value. The novel US technique NLV is easy for fast screening exam for MAFLD patients with good diagnostic results. We expect future application of this technology for diffuse liver disease evaluation.
